# Enaminone Modulators of Extrasynaptic α_4_β_3_δ γ-Aminobutyric Acid_A_ Receptors Reverse Electrographic Status Epilepticus in the Rat After Acute Organophosphorus Poisoning

**DOI:** 10.3389/fphar.2019.00560

**Published:** 2019-05-24

**Authors:** Timothy B. C. Johnstone, Hilary S. McCarren, Jay Spampanato, F. Edward Dudek, John H. McDonough, Derk Hogenkamp, Kelvin W. Gee

**Affiliations:** ^1^Department of Pharmacology, School of Medicine, University of California, Irvine, Irvine, CA, United States; ^2^Neuroscience Department, Medical Toxicology Research Division, United States Army Research Institute of Chemical Defense, Aberdeen, MD, United States; ^3^Department of Neurosurgery, University of Utah School of Medicine, Salt Lake City, UT, United States

**Keywords:** diisopropyl fluorophosphate, soman, nerve agent, midazolam, extrasynaptic, γ-aminobutyric acid-A receptors

## Abstract

Seizures induced by organophosphorus nerve agent exposure become refractory to treatment with benzodiazepines because these drugs engage synaptic γ-aminobutyric acid-A receptors (GABA_A_Rs) that rapidly internalize during *status epilepticus* (SE). Extrasynaptic GABA_A_Rs, such as those containing α_4_β_3_δ subunits, are a putative pharmacological target to comprehensively manage nerve agent-induced seizures since they do not internalize during SE and are continuously available for activation. Neurosteroids related to allopregnanolone have been tested as a possible replacement for benzodiazepines because they target both synaptic and extrasynaptic GABA_A_Rs receptors. A longer effective treatment window, extended treatment efficacy, and enhanced neuroprotection represent significant advantages of neurosteroids over benzodiazepines. However, neurosteroid use is limited by poor physicochemical properties arising from the intrinsic requirement of the pregnane steroid core structure for efficacy rendering drug formulation problematic. We tested a non-steroidal enaminone GABA_A_R modulator that interacts with both synaptic and extrasynaptic GABA_A_Rs on a binding site distinct from neurosteroids or benzodiazepines for efficacy to control electrographic SE induced by diisopropyl fluorophosphate or soman intoxication in rats. Animals were treated with standard antidotes, and experimental therapeutic treatment was given following 1 h (diisopropyl fluorophosphate model) or 20 min (soman model) after SE onset. We found that the enaminone 2-261 had an extended duration of seizure termination (>10 h) in the diisopropyl fluorophosphate intoxication model in the presence or absence of midazolam (MDZ). 2-261 also moderately potentiated MDZ in the soman-induced seizure model but had limited efficacy as a stand-alone anticonvulsant treatment due to slow onset of action. 2-261 significantly reduced neuronal death in brain areas associated with either diisopropyl fluorophosphate- or soman-induced SE. 2-261 represents an alternate chemical template from neurosteroids for enhancing extrasynaptic α_4_β_3_δ GABA_A_R activity to reverse SE from organophosphorous intoxication.

## Introduction

Acute organophosphorus poisoning is a military and civilian threat that causes a complex cascade of lethal central and peripheral nervous system toxicities. Organophosphorus nerve agents are related to organophosphorus insecticides and include chemicals such as soman, sarin, cyclosarin, tabun, VX, and VR (Smart, 1997; Balali-Mood and Balali-Mood, 2008; Worek et al., 2016). Organophosphorus nerve agents functionally inactivate acetylcholinesterase, the enzyme responsible for clearing the neurotransmitter acetylcholine from nerve synapses (Balali-Mood and Balali-Mood, 2008; Worek et al., 2016). Organophosphorus nerve agents cause levels of acetylcholine to accumulate and remain at synapses, leading to overstimulation of peripheral and central muscarinic/nicotinic receptors (McDonough and Shih, 1997; Watson et al., 2009). The resulting “cholinergic storm” can precipitate SE and culminate in death (Jokanović and Kosanović, 2010).

Standard medical countermeasures for organophosphorus-induced seizures include a clinically-used anticonvulsant benzodiazepine to promote global GABA_A_R mediated inhibition in the brain to interrupt SE and limit the resulting neuropathology (Shih et al., 2003). However, benzodiazepines exhibit a time-dependency for efficacy to stop SE caused in part by the internalization of post-synaptic GABA_A_Rs (Naylor et al., 2005; McDonough et al., 2009, 2010). Soman exposure is reported to elicit reduced GABA_A_R function in rat brain (Lallement et al., 1993) and reduced surface expression of GABA_A_Rs specifically in the hippocampus (Wang et al., 2011). *In vitro* studies with soman in hippocampal slices suggest that a 30% reduction of surface expression of GABA_A_Rs renders hippocampal neurons unresponsive to diazepam (Wang et al., 2011). These remaining GABA_A_Rs may be extrasynaptic receptors responsible for setting the background tone of global neuronal inhibition (Farrant and Nusser, 2005; Chuang and Reddy, 2018). Extrasynaptic α_4_β_3_δ GABA_A_R subunits do not internalize by endocytosis after refractory SE (Goodkin et al., 2008), nor do they desensitize in continuous seizure models like synaptic GABA_A_Rs (Zhan and Nadler, 2009). Therefore, extrasynaptic GABA_A_Rs could be a critical pharmacological target to control soman-induced SE since benzodiazepines are almost inactive at GABA_A_R subtypes containing δ-subunits (Reddy, 2016; Reddy and Estes, 2016; Chuang and Reddy, 2018) and depend on the presence of synaptic γ-subunits for GABA_A_R receptor binding and modulation (Pritchett et al., 1989).

The engagement of synaptic and extrasynaptic GABA_A_Rs by neurosteroids could theoretically produce a more global enhancement of inhibitory neurotransmission (Kokate et al., 1998; Reddy and Rogawski, 2000; Lambert et al., 2009). For example, the endogenous neurosteroid allopregnanolone is active in multiple seizure models and shows clinical activity against benzodiazepine-resistant SE (Belelli et al., 1989; Rogawski et al., 2013; Vaitkevicius et al., 2013; Broomall et al., 2014). Allopregnanolone shows prolonged activity after exposure to convulsant agents, unlike MDZ and other benzodiazepines (Lossin et al., 2013). Unfortunately, poor aqueous solubility has delayed the clinical development of prototypical natural and synthetic neurosteroids for epilepsy despite > 20 years of clinical testing, compelling preclinical efficacy, and an attractive safety profile (Lossin et al., 2013; Rogawski et al., 2013; Vaitkevicius et al., 2013; Broomall et al., 2014). Nonetheless, recently described neurosteroids like SGE-516 have shown promising utility to terminate SE from organophosphorus-induced intoxication (Martinez-Botella et al., 2015; Althaus et al., 2017).

We recently reported 2-261 as a prototype non-steroid small molecule with neurosteroid-like activity at extrasynaptic α_4_β_3_δ GABA_A_Rs similar to allopregnanolone (Johnstone et al., 2019) or ganaxolone (unpublished observations). 2-261 and related enaminone modulators are non-steroidal small molecules that modulate synaptic GABA_A_Rs similar to neurosteroids (Hogenkamp et al., 2007; Gee et al., 2010; Yoshimura et al., 2014). 2-261 binds to a distinct site on GABA_A_Rs and is functionally coupled to neurosteroid and benzodiazepine sites by positive heterotropic cooperativity (Hogenkamp et al., 2007). The topological separation of drug binding sites on the receptor offers the potential for additive or synergistic effects when combined with benzodiazepines due to allosteric changes in receptor conformation. 2-261 and related enaminones display unique pharmacological properties that can be manipulated to produce desirable side effect/safety profiles based on receptor subunit-selectivity not possible with other classes of GABA_A_R modulators (Gee et al., 2010). For example, 2-261 retains anxiolytic activity without ataxia, sedation, cognitive impairment, rewarding effects (linked to addiction), tolerance or withdrawal (Yoshimura et al., 2014). The mitigation of specific side effects like ataxia and sedation may be a competitive advantage relative to other positive allosteric modulator strategies (e.g., neurosteroids) to enhance GABA_A_R efficacy as a medical countermeasure strategy. Therefore, 2-261 was tested in the National Institutes of Health CounterACT Neurotherapeutic Screening Program using validated rat diisopropyl fluorophosphate and soman exposure protocols to demonstrate proof-of-concept efficacy of 2-261 to terminate organophosphorus-induced seizure activity and/or prevent neuronal loss in selected brain regions susceptible to the toxic effects of organophosphorus compounds.

## Materials and Methods

### Synthesis of 2-261

2-261 (2-chloro-α-[[4-(chlorophenyl)amino]methylene]-*N*-[(1*S*)-1-methylpropyl]-β-oxobenzenepropanamide) was synthesized as previously given (Hogenkamp et al., 2007).

### Animals

Male Sprague-Dawley rats (50–75 g, diisopropyl fluorophosphate studies; 250–300 g, soman studies) were purchased from Charles River Laboratories (Wilmington, MA, United States). Upon arrival, the rats were housed in a temperature-controlled vivarium on a 12-h light: 12-h dark cycle, with *ad libitum* access to food and water throughout the study. All surgical and experimental procedures were reviewed and approved by the University of Utah or United States Army Medical Research Institute of Chemical Defense Institutional Animal Care and Use Committee.

### Electroencephalographic (EEG) Implant Surgery

EEG recording electrodes were implanted in rats anesthetized with isoflurane (3–5% for induction, 1–3% for maintenance) and placed in a stereotaxic instrument. Bupivacaine (2.5%) was injected intradermally in the scalp on either side of the intended incision site. To implant the electrodes, a longitudinal midline incision was made in the scalp, which was then retracted laterally to expose the skull. Next, burr holes were drilled through the skull for placement of screw electrodes that touched the surface of the cortex. In the diisopropyl fluorophosphate model, two electrodes were placed on the right side of the midline, and a ground electrode was positioned on the left side of the skull. In the soman model, an electrode was placed over each hemisphere of the parietal cortex, and a ground electrode was placed over cerebellum. The headset was then secured in place with dental cement. Finally, the wound was closed with sutures, and rats were returned to their home cages for at least 5 days prior to testing.

### Diisopropyl Fluorophosphate Exposure

Following the recovery period, the conscious, unrestrained rats (150 and 225 g at the time of testing) were tested in individual Plexiglas cages. The implanted electrodes were connected to spring-covered EEG cables (Plastics One, Roanoke, VA, United States) for recording. The protocol used a delayed-treatment rodent model of organophosphorus exposure that induced SE following administration of diisopropyl fluorophosphate. All rats were given pyridostigmine bromide [0.026 mg/kg, intramuscular (i.m.)] 30 min prior to diisopropyl fluorophosphate [4.0–6.0 mg/kg, subcutaneous (s.c.)], and atropine methyl nitrate (2 mg/kg, i.m.) plus 2-pyridine aldoxime methyl chloride (25 mg/kg, i.m.) 1 min following diisopropyl fluorophosphate. These antidote compounds were necessary to decrease mortality resulting from the peripheral, potentially lethal effects of diisopropyl fluorophosphate, to ensure survival of a sufficient number of rats for collection and evaluation of EEG data. After diisopropyl fluorophosphate administration, EEG recordings were directly observed and the time of the initial electrographic seizure noted. Exactly 60 min after the initial EEG seizure response, rats were administered MDZ (1.78 mg/kg, i.m.) and vehicle (intraperitoneal), MDZ plus the test compound (intraperitoneal), MDZ alone, or the test compound alone. EEG activity was then recorded continuously for the 24 h following diisopropyl fluorophosphate administration. On the day following treatment, rats were euthanized for tissue collection.

### Soman Exposure

At approximately 07:00 on experiment day, the rats (between 300 and 375 g) were placed in individual recording chambers and connected to the EEG recording system via their headpiece plug. Following 60 min of normal baseline EEG recording, rats were pretreated with 125 mg/kg asoxime chloride, delivered intraperitoneally. Thirty minutes after pretreatment, 180 μg/kg of the nerve agent soman was delivered subcutaneously. This dose elicited EEG seizure activity in 100% of rats studied. One minute after soman challenge, rats received 2 mg/kg atropine methyl nitrate i.m. Along with asoxime chloride, atropine methyl nitrate protected rats from systemic toxicity of soman to allow survival until development of neurological symptoms. Onset of EEG seizure activity was defined by the appearance of repetitive spikes and sharp waves with an amplitude greater than twice that of the baseline and a duration of at least 10 s. At 20 min after seizure onset, rats were treated with 0.45 mg/kg atropine sulfate admixed with 25 mg/kg 2-pyridine aldoxime methyl chloride (i.m.). They also received 1.8 mg/kg MDZ (i.m.) and saline (intraperitoneal), MDZ plus the test compound (intraperitoneal), or the test compound alone. In order to minimize the number of rats subjected to an ineffective treatment regimen, no rats were treated with MDZ alone. Following administration of the test compound, EEG activity was recorded for at least 4 h. At the end of the 4-h recording period, the EEG record of each subject was visually evaluated for continuing evidence of seizure activity. Rats were then euthanized for collection of brain tissue.

### Electroencephalogram Recording and Analysis

In the diisopropyl fluorophosphate model, EEG signals were amplified with EEG100 amplifiers (high-pass filter, 1 Hz, low-pass, 100 Hz, notch filter at 60 Hz, 5000x gain), then digitized at 500 Hz using an MP150 analog-to-digital converter, and recorded with AcqKnowledge software (BioPac Systems, Incorporated Santa Barbara, CA, United States) for subsequent analysis. In the soman model, EEG signals were amplified with 1902 amplifiers, digitized with a Micro1401 data acquisition interface, and recorded with Spike2 software (all from Cambridge Electronic Design Limited, Cambridge, United Kingdom). Data channels were sampled at 512 Hz and digitally filtered with a high-pass 0.3 Hz filter, a low-pass 100 Hz, and a 60 Hz notch filter. Power spectral density in the frequency range of 20–60 Hz (gamma) (Lehmkuhle et al., 2009), and spike rate (White et al., 2006) were calculated from the raw EEG recordings using custom Python-based software. Changes in the mean power in the gamma band and mean spike rate frequency during SE were determined by subtracting baseline levels of both variables measured during a 10 min time bin prior to chemoconvulsant administration from values observed during sequential 15-min bins (diisopropyl fluorophosphate model) or 5-min bins (soman model) during seizure activity. The mean changes in gamma power and spike rate frequency, and the 95% confidence intervals were calculated.

### EEG Statistics

Mean changes from baseline in EEG spike rate frequency and gamma band power for each experimental group were compared at specific time points (30 min before chemoconvulsant, at treatment time, and then hourly for the remainder of the analysis period). To determine if differences between groups at each hour were statistically significant, unpaired *t*-tests were used because they provided periodic information about instantaneous differences in seizure activity between groups. This approach was chosen rather than a repeated-measures two-way ANOVA (time × treatment group) because time always accounts for the majority of variation (i.e., spike frequency and gamma power always increase dramatically between baseline and SE onset regardless of treatment), which can obscure the ability to detect meaningful but lower magnitude differences between groups post-treatment.

### Tissue Preparation

Following the EEG recording session, rats were deeply anesthetized with isoflurane (diisopropyl fluorophosphate model) or sodium pentobarbital (soman model). They were then transcardially perfused with saline until fully exsanguinated, followed by 10% formalin for tissue fixation. For the diisopropyl fluorophosphate model, brains were subsequently placed in 30% sucrose for cryoprotection. Brains were then flash frozen and 30 μm coronal sections were cut between bregma coordinates −1.8 to −6.3 mm (Paxinos and Watson, 2006). For the soman model, brains were embedded in paraffin and sectioned into 5 μm coronal slices. The section corresponding to 3.24 mm posterior to bregma (Paxinos and Watson, 2006) was collected. All brain sections were mounted on glass slides and stained with FluoroJade B (Histo-chem, Inc., Jefferson, AR, United States) using previously described techniques (Schmued and Hopkins, 2000). Individual sections were imaged with a Hamamatsu Nanozoomer 2.0 HT (diisopropyl fluorophosphate model) or an Olympus VS120-L100-W virtual slide microscope (soman model) and VS-ASW software (Olympus Corporation, Tokyo, Japan).

### FluoroJade B Neuron Counting in Diisopropyl Fluorophosphate Model

An unbiased random-sampling technique to quantify the number of FluoroJade B positive neurons in selected brain sections was used to estimate the neuropathological effects of diisopropyl fluorophosphate induced SE. Specifically, four sections/slides from each brain area, separated by a minimum of 60 μm, were selected for evaluation. Anatomical markers were used to defined brain regions in correspondence with the Rat Brain in Stereotaxic Coordinates by George Paxinos as follows: for Parietal, dCA1, dCA3, Hilus, Thalamus, Amygdala and Piriform Cortex plate #30 “Bregma −3.14 mm” and for vCA1, vCA3 and Entorhinal Cortex plate #37 “Bregma −4.80 mm”. For areas with uniform distribution of neurons (thalamus, amygdala, piriform cortex, parietal cortex, and entorhinal cortex), a grid of 175-μm × 175-μm squares was randomly placed over the entire image. The image was rotated when necessary to align the laminar architecture of the region parallel with one direction of the grid. The counting region of interest was then demarcated by the counter and required to include a pre-set number of squares (see Table 1) defined by the area of each brain region of interest. For areas with non-uniform distribution of neurons and curved architectures (dorsal *cornu ammonis* 1, dorsal *cornu ammonis* 3, ventral *cornu ammonis* 1, ventral *cornu ammonis* 3, and hilus), the counter first aligned the preset number of boxes with each region. Then, based on a set of defined rules for each brain region, the counter may have readjusted several boxes of the counting region of interest to better map the curvature of the principal cell layer based on the mandatory sizes for counting regions of interest.

**Table 1:** Mandatory counting region of interest sizes.

Dorsal *cornu ammonis* 1	12 boxes to fit
Dorsal *cornu ammonis* 3	3x3 grid
Hilus	18 boxes to fit
Amygdala	3x3 grid
Thalamus	4x4 grid
Piriform cortex	4x4 grid
Parietal cortex	5x5 grid
Entorhinal cortex	6x5 grid
Ventral *cornu ammonis* 1	12 boxes to fit
Ventral *cornu ammonis* 3	12 boxes to fit

This was sometimes necessary because the principal cell layer has both the highest density of neurons in the *cornu ammonis* regions of the hippocampus, as well as the highest density of FluoroJade B labeled neurons following diisopropyl fluorophosphate induced SE. Then, within each of these selected sections, three of the 175 μm x 175 μm square counting boxes were chosen by a random number generator, and the blinded observer counted the number of FluoroJade B positive neurons in each of those three boxes. The brain regions selected for analysis were the dorsal and ventral hippocampus (*cornu ammonis* 1, *cornu ammonis* 3), hilus, amygdala, thalamus, and the parietal, piriform, and entorhinal cortices. These sites were selected for this study as they have previously been shown to mediate the initiation, propagation, and/or maintenance of seizure activity (Gale, 1992; Myrher, 2007; Myhrer et al., 2008; Apland et al., 2010; Skovira et al., 2010, 2012). A treatment-blinded technician counted the number of FluoroJade B-expressing cells in each cropped region.

### FluoroJade B Neuron Counting in Soman Model

FIJI was used to crop out regions of interest for neuropathology using the following dimensions (width × height): amygdala (1000 μm × 1000 μm), piriform cortex (200 μm × 2000 μm), thalamus (1000 μm × 1300 μm), and parietal cortex (1500 μm × 2000 μm). The hippocampus counting region was defined by the anatomical boundaries of the structure. A treatment-blinded technician counted the number of FluoroJade B-expressing cells in each cropped region.

### Neuropathology Statistics

FluoroJade B positive neuron counts for each region of interest were averaged for each treatment and compared by unpaired *t*-test. For all statistical evaluations, *p* < 0.05 was considered significant.

## Results

**FIGURE 1:**
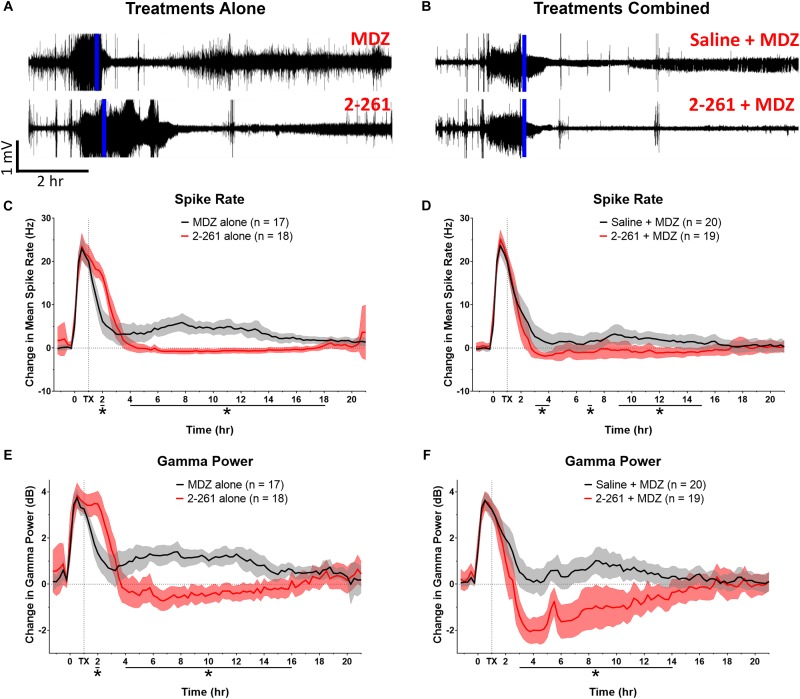
Effect of treatment by 2-261 alone or combined with MDZ after diisopropyl fluorophosphate intoxication. Representative example of EEG recordings from rats challenged with diisopropyl fluorophosphate and treated 60 min after the onset of SE (indicated by vertical blue line) with either 1.8 mg/kg MDZ i.m. alone or 120 mg/kg 2-261 i.p. alone **(A)**. EEG traces in **(B)** show rats treated with saline combined with MDZ or 2-261 combined with MDZ. Summary of the change in mean spike frequency and gamma power after 2-261 treatment alone **(C,E)** or combined with MDZ **(D,F)** after diisopropyl fluorophosphate exposure. Asterisks indicate time points at which there was a significantly different response between treatment groups in spike rate or gamma power as measured by *t*-test, *N* = 17–20 rats for each treatment group. MDZ, midazolam; hr, hour; mV, millivolts; Hz, hertz; dB, decibels.

**FIGURE 2:**
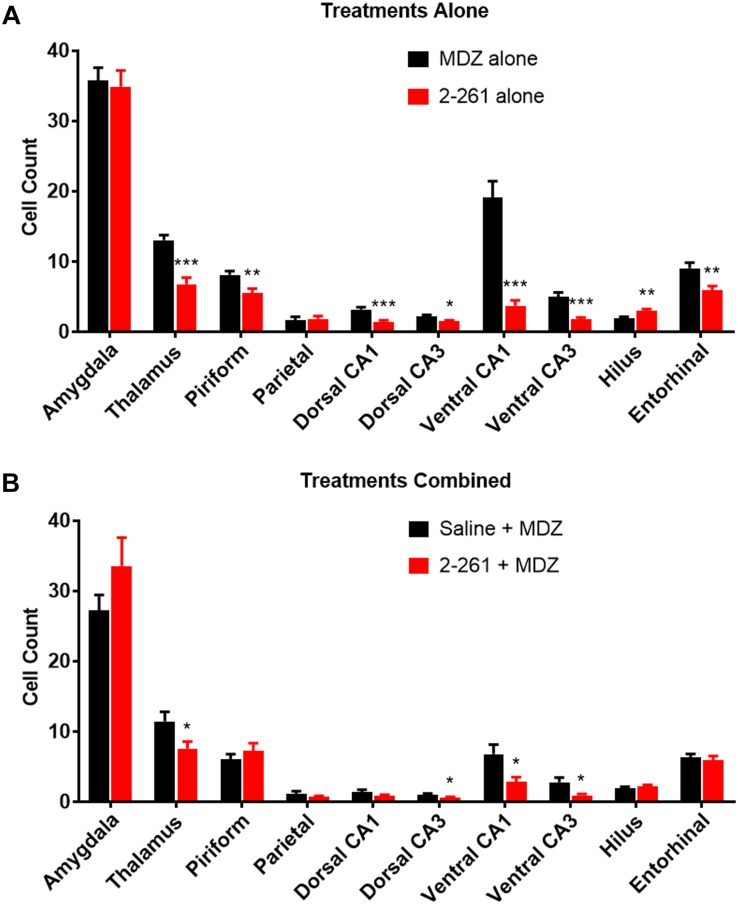
Effect of treatment by 2-261 alone **(A)** or combined with MDZ **(B)** on Fluoro-Jade B labeled neurons in various brain regions removed from subject rats 24 h after receiving diisopropyl fluorophosphate challenge. Asterisks indicate brain regions in which there was a significantly different response (^∗^*p* < 0.05, ^∗∗^*p* < 0.01, ^∗∗∗^*p* < 0.001) between treatment groups in the number of Fluoro-Jade B stained neurons as measured by *t*-test, *N* = 17–20 rats for each treatment group. MDZ, midazolam.

The non-steroidal enaminone 2-261 had a delayed anti-seizure effect in the diisopropyl fluorophosphate model and reduced subsequent neuronal death with or without MDZ. We administered 120 mg/kg 2-261 (intraperitoneal) to rats 60 min after acute diisopropyl fluorophosphate intoxication compared to the standard medical countermeasure MDZ. 2-261 produced an anti-seizure effect as measured by lower spike frequency and gamma-band power relative to baseline than that seen in rats treated with MDZ alone (Figure 1A,C,E). The initial anti-seizure effect of 2-261 was observed to occur approximately 1 h later than MDZ, but produced significant reductions of spike frequency and gamma power from hours 4 to 16 (Figure 1C,E). At 120 mg/kg intraperitoneal, 2-261 also enhanced the efficacy of MDZ to reduce epileptiform discharge, gamma power, and mean spike frequency to below baseline levels (Figure 1B,D,F). However, 2-261 was ineffective on EEG measures when tested at 60 mg/kg intraperitoneal alone or in combination with MDZ after diisopropyl fluorophosphate intoxication (data not shown). 2-261 alone significantly reduced neuronal death as measured by FluoroJade B staining relative to MDZ alone in piriform cortex, thalamus, entorhinal cortex, and multiple areas of the hippocampus, consistent with the long duration of anticonvulsant effect on the EEG (Figure 2A). 2-261 did not cause reductions in FluoroJade B staining in the parietal cortex or amygdala and slightly enhanced FluoroJadeB staining in the hilus (Figre 2A). In the presence of MDZ, 2-261 only showed reductions in FluoroJade B staining in *cornu ammonis* 1/*cornu ammonis* 3 hippocampal areas and thalamus (Figure 2B). The 2-261 + MDZ results are consistent with the observation that compounds like 2-261 bind a different receptor site on GABA_A_Rs and exhibit positive heterotropic cooperativity because of allosteric coupling to the binding site for benzodiazepines (Hogenkamp et al., 2007). This also indicates that extrasynaptic γ-aminobutyric acid-A receptors are engaged simultaneously with synaptic GABA_A_Rs when 2-261 is administered.

**FIGURE 3:**
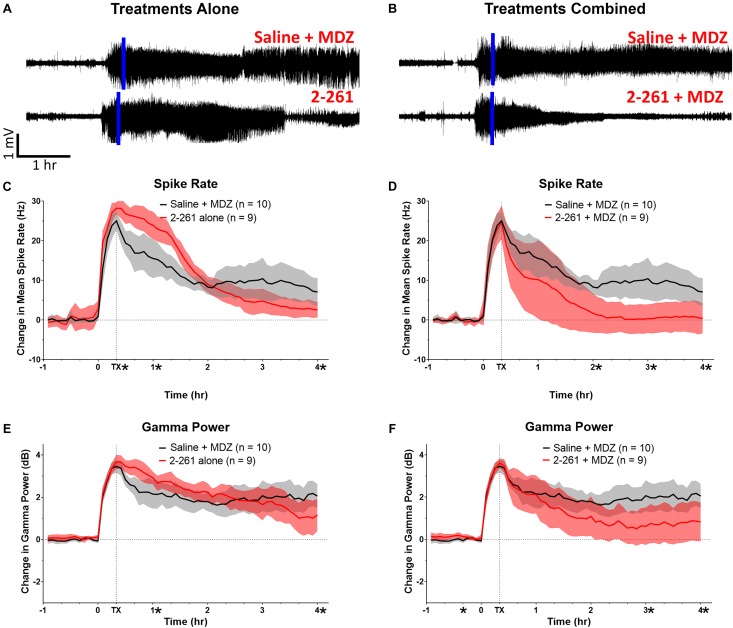
Effect of treatment by 2-261 alone or combined with MDZ after soman intoxication. Representative example of EEG recordings from subject rats after soman challenge and treated 20 min after the onset of SE (indicated by vertical blue line) with either saline plus 1.8 mg/kg MDZ i.m. or 120 mg/kg 2-261 i.p. alone **(A)**. EEG traces in **(B)** show rats treated with saline combined with MDZ or 2-261 combined with MDZ. Summary of the change in gamma power and mean spike frequency after 2-261 treatment alone **(C,E)** or combined with MDZ **(D,F)** after soman exposure. Asterisks indicate time points at which there was a significantly different response between treatment groups in spike rate or gamma power as measured by *t*-test, *N* = 9–10 rats for each treatment group. MDZ, midazolam; hr, hour; mV, millivolts; Hz, hertz; dB, decibels.

**FIGURE 4:**
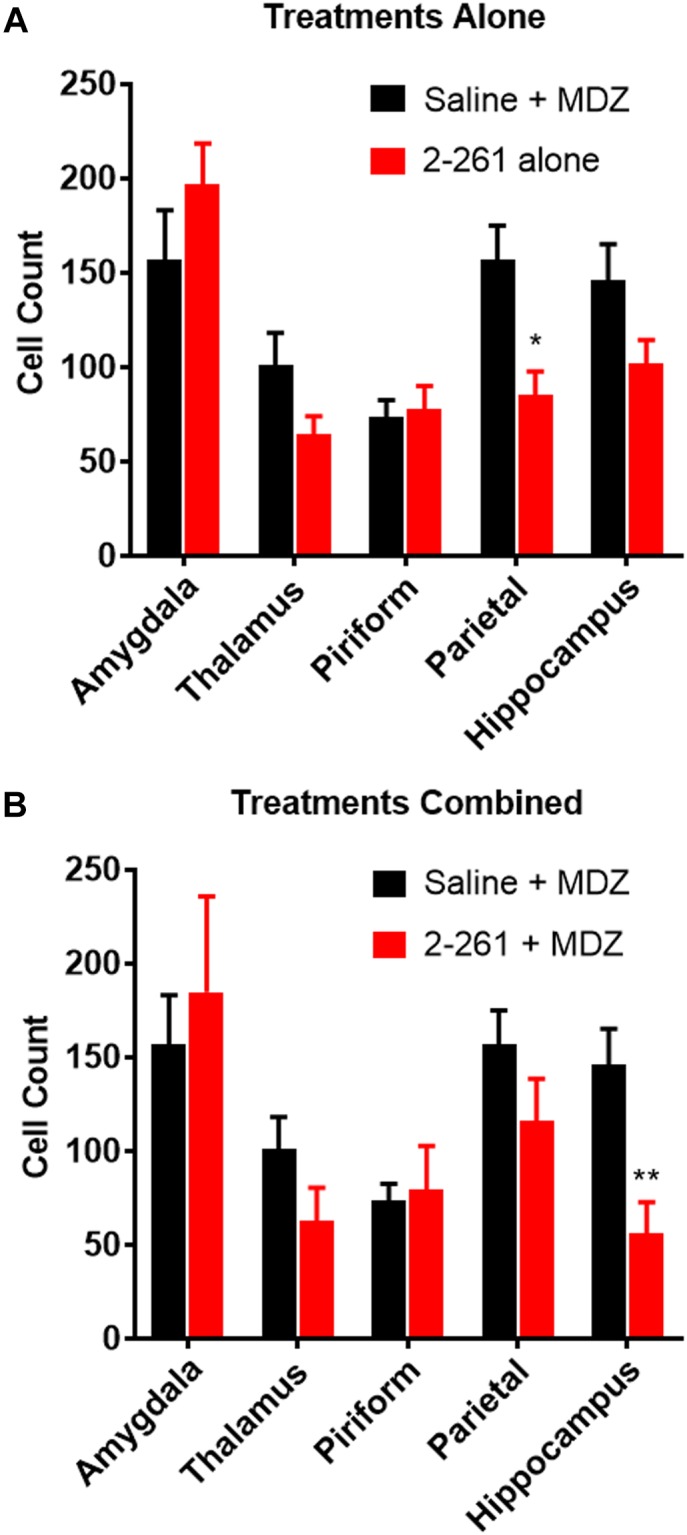
Effect of treatment by 2-261 alone **(A)** or combined with MDZ **(B)** on Fluoro-Jade B labeled neurons in various brain regions removed from subject rats 4 h after receiving soman (180 μg/kg, s.c.) challenge. Asterisks indicate brain regions in which there was a significantly different response (^∗^*p* < 0.05, ^∗∗^*p* < 0.01) between treatment groups in the number of Fluoro-Jade B stained neurons as measured by *t*-test, *N* = 9–10 rats for each treatment group. MDZ, midazolam.

2-261 showed modest changes in seizure activity in the soman model and reduced soman-initiated neuronal death in the presence or absence of MDZ. 2-261 was ineffective on EEG measures when tested at 60 mg/kg intraperitoneal in combination with MDZ after soman intoxication (data not shown). However, the 120 mg/kg intraperitoneal dose of 2-261 active in the diisopropyl fluorophosphate model showed no immediate efficacy to reduce gamma power or mean spike frequency after soman intoxication compared to MDZ-treated rats until the time when recordings were terminated, 4 h (Figure 3A,C,E). This is consistent with the lack of significant reductions in FluoroJade B staining at 4 h post-treatment, with the exception of the parietal cortex (Figure 4A). These observations are also consistent with the slow onset observed in the diisopropyl fluorophosphate model and suggest 2-261 does not produce sufficiently rapid efficacy as a stand-alone anti-seizure therapy when administered at 120 mg/kg in the absence of another anticonvulsant. 2-261 does facilitate the maximum MDZ-induced reduction of EEG spiking (Figure 3B), relative spike frequency (Figure 3D) and, to a lesser extent, the relative gamma power (Figure 3F) up to 4 h after soman exposure. 2-261 showed significant changes in neuronal death in the hippocampus when combined with MDZ after soman exposure, but not in other brain regions evaluated (Figure 4A,B). These small changes with 2-261 most likely reflect the limited activity seen on the electroencephalogram recordings and correlate with the inability to terminate seizures by 4 h post-soman exposure.

## Discussion

The utility of modulating extrasynaptic GABA_A_Rs in a therapeutically useful way to treat SE caused by organophosphosphorus nerve agents is currently limited to a handful of chemical classes. Orthosteric agonists of extrasynaptic GABA_A_Rs such as gabaxodol (Orser, 2006) and allosteric modulators of extrasynaptic GABA_A_Rs like the delta-selective (DS) compounds (Wafford et al., 2009; Jensen et al., 2013) or neurosteroids (Reddy, 2016; Althaus et al., 2017) may be able to reverse organophosphorous nerve agent-induced SE. However, the practical utility of many of these classes of extrasynaptic GABA_A_R modulators is limited by the inherent sedative effects produced after administration. While this sedative/hypnotic state will likely reduce gamma power or spike rate and halt seizure progression in a hospital setting for certain periods of time, there is questionable benefit to soldiers on the battlefield who need to remain at least conscious enough to be ambulatory for extraction to a hospital setting. Therefore, we tested whether a non-sedating enaminone, 2-261 (Gee et al., 2010; Yoshimura et al., 2017), could be an alternative to other extrasynaptic GABA_A_Rs modulators to reverse organophosphosphorus nerve agent-induced SE.

Our results suggest that the enaminone GABA_A_R modulator, 2-261, can reduce electrographic seizures and block neuronal death caused by acute organophosphosphorus poisoning either alone or in combination with the benzodiazepine, MDZ. The sustained (∼12 h) activity of 2-261 alone in the diisopropyl fluorophosphate model suggests that high-efficacy modulators of extrasynaptic α_4_β_3_δ GABA_A_Rs have utility for the treatment of SE following organophosphosphorus poisoning, an observation also supported by the use of NSs to terminate organophosphosphorus-induced SE (Reddy, 2016; Althaus et al., 2017). 2-261 enhances MDZ efficacy when the two drugs are combined for treatment against diisopropyl fluorophosphate-induced seizures and displays a long duration of action with or without MDZ. The termination of the diisopropyl fluorophosphate-induced electrographic seizures by 2-261 results in reductions in neuronal death in brain areas susceptible to SE, such as the hippocampus. However, the 2-261 effect in the diisopropyl fluorophosphate model is limited by a late onset of drug activity that is not observable until >3 h post-treatment. A potential absorption issue due to poor solubility of 2-261 at the doses tested limits the onset of the anti-seizure effect as indicated by the lack of full efficacy at early time points despite a long duration of action. This explains why brain areas such as the amygdala, parietal cortex, and hilus show no protection from neuronal death relative to MDZ alone. Brain regions like the amygdala are the most severely damaged areas of the brain after nerve-agent induced SE (Shih et al., 2003; Baille et al., 2005; Aroniadou-Anderjaska et al., 2009; Apland et al., 2010) due to their exceptionally high level of cholinergic innervation (Mesulam et al., 1992). Notably, the neurosteroid SGE-516 was very efficacious at reducing apparent neuronal death in the amygdala in the soman model whereas 2-261 did not show any trend for neuroprotection in this particular brain region for either diisopropyl fluorophosphate or soman intoxication (Althaus et al., 2017). This may be due to the delayed onset by 2-261 relative to SGE-516, or it could be due to additional neuroprotective mechanisms of neurosteroids not shared by enaminones. For example, progesterone shows neuroprotection, myelin formation, anti-inflammatory, and neurogenesis effects that are exhibited in various models of neurodegeneration, traumatic brain injury, and spinal cord transection (Schumacher et al., 2014). These protective effects likely arise from improvements to mitochondrial function including optimized mitochondrial dynamics, metabolic pathways and ultrastructure, outcomes directly related to stimulating neurosteroidogenesis (De Nicola et al., 2018).

The effect of delayed onset of 2-261 activity due to impaired pharmacokinetics is readily apparent in the soman-induced SE model. 2-261 without MDZ shows no significant reversal of soman-induced electrographic seizures until well after the third hour post-treatment, an observation also seen in the diisopropyl fluorophosphate model. Since the soman model only measures the EEG recordings for 4 h there may be insufficient levels at earlier time points to extensively activate extrasynaptic GABA_A_Rs with 2-261, and hence no actual seizure block is observed. 2-261 compares favorably to SGE-516 in the soman model to reduce electrographic seizures but the potency of SGE-516 was 10-fold greater than 2-261, and any sedative effects of SGE-516 were not reported at this dose (Martinez-Botella et al., 2015; Althaus et al., 2017). The absence of 2-261 activity early in the soman-induced seizure model is consistent with the data obtained in the diisopropyl fluorophosphate model and is a result of a slow onset of action. The SE and subsequent lethality produced by soman intoxication is extremely rapid and requires an anticonvulsant countermeasure with an equally fast onset of action since survivability is unlikely in the absence of early reductions in EEG markers of epileptiform activity. In this regard, enaminone modulators would have greater efficacy against soman seizures if physicochemical properties of the prototype 2-261 were optimized. Ideally, an enaminone modulator with reduced lipophilicity should improve solubility to enhance onset of action and duration of action.

The DFP/soman animal studies were modeled in a manner similar to a clinical trial utilizing an active control group whereby one group receives the standard of care and a second group is administered the experimental drug (Evans, 2010). A placebo control animal group administered soman or DFP without MDZ as an anticonvulsant was excluded from this study and MDZ was the active control instead. It is not possible to perform OP/NA-induced clinical trials in humans so there exists a specific need to model clinical trial designs in rodents in order to identify non-inferior or superior treatments to standard-of-care that is relevant to drug discovery. Therefore the primary question in this study relates to 2-261 treatment of OP/NA-induced SE and the standard-of-care treatment, MDZ. MDZ is the appropriate benchmark by which to compare 2-261 in this study since an ideal novel countermeasure should match or exceed the efficacy of currently available therapies. The dose of MDZ used in this study is the rat-scaled equivalent of 20 mg MDZ in a 70 kg adult human. This is twice the dose of MDZ found to effectively terminate SE in the RAMPART study and also represents the current guidelines for pre-hospital MDZ dosing following nerve agent exposure (Silbergleit et al., 2012). This MDZ dose was chosen because it demonstrates that both the DFP and soman models are benzodiazepine resistant based on clinical standards. It is also a realistic approximation of what would be employed for a human OP casualty. It is possible that increasing the dose of MDZ employed in these models would lead to more optimal seizure control. However, higher doses of MDZ cause increased sedation and a higher risk of respiratory complications, both of which are detrimental in civilian or military emergency settings.

Placebo animal groups were also omitted from this study for other additional reasons. In the DFP model it is reported that untreated DFP-induced SE does not spontaneously remit. DFP-induced SE follows a very well-known gradual decay to baseline over a period of 10 h, an observation that makes the likelihood low of a type I error by excluding a placebo control group when concluding any effect of treatment (Pouliot et al., 2016). Exclusion of a placebo group in this study is also based on ethical issues related to animals subjected to unremitting SE for 24 h, especially given that placebo control data for the DFP model has been previously published (Pouliot et al., 2016). These previously reported data effectively serve as historical controls to the present studies since the current study design and data analysis were identically performed to these previous studies. Ethical use of animals also confounds inclusion of placebo controls in the soman-induced SE model because untreated animals die at a rate of >90%. High mortality in this model would demand an undesirable number of animals to achieve acceptable statistical significance and power.

Our data on 2-261 combined with MDZ after diisopropyl fluorophosphate or soman exposure illustrates that topological separation of drug binding sites on GABA_A_Rs can provide additive effects since changes in EEG recordings were greater in the presence of all concentrations of 2-261 tested. These findings would be expected because 2-261 binds to a distinct site on GABA_A_Rs that is functionally coupled to neurosteroid and benzodiazepine sites by positive heterotropic cooperativity (Hogenkamp et al., 2007). Enaminones also display unique pharmacological properties that can be manipulated to improve side effect/safety profiles based on receptor subunit-selectivity not possible with other known classes of GABA_A_R modulators (Gee et al., 2010). For example, 2-261 retains anxiolytic activity without ataxia, sedation, cognitive impairment, rewarding effects (linked to addiction), tolerance or withdrawal (Yoshimura et al., 2014). The mitigation of specific side effects like ataxia and sedation is an obvious advantage relative to other positive allosteric modulator strategies (e.g., neurosteroids) for modulating GABA_A_Rs since motor skills are necessary to facilitate evacuation following mass organophosphosphorus poisoning.

The strategy of targeting extrasynaptic α_4_β_3_δ γ-aminobutyric acid-A receptors to counteract SE produced by acute organophosphosphorus poisoning is a viable pharmacological method validated by evidence from two separate classes of GABA_A_R allosteric modulators distinct from benzodiazepines. Both the neurosteroid and enaminone approaches are good starting points for developing a rapid-acting countermeasure that can enhance the utility of MDZ or operate as standalone replacements to benzodiazepines. However, both chemical classes have specific issues that require resolution before further development of either can occur as treatment for organophosphosphorus poisoning.

## Ethics Statement

The study was carried out in accordance with the Guide for the Care and Use of Laboratory Animals (National Research Council, 2011), and the Animal Welfare Act of 1966 (P.L. 89-544), as amended. The protocol was approved by the Animal Care and Use Committee at the United States Army Medical Research Institute of Chemical Defense (GD Animal Model) and the University of Utah (DFP Animal Model).

## Author Contributions

DH and TJ synthesized 2-261. JS analyzed the data from animal experiments with diisopropyl fluorophosphate. HM performed and analyzed the data from animal experiments with soman. TJ, HM, and JS drafted the manuscript. JM, FD, and KG supervised the work. All authors discussed results and commented on the manuscript.

## Conflict of Interest Statement

The authors declare that the research was conducted in the absence of any commercial or financial relationships that could be construed as a potential conflict of interest.
